# Novel Viruses That Lyse Plant and Human Strains of *Kosakonia cowanii*

**DOI:** 10.3390/v13081418

**Published:** 2021-07-21

**Authors:** Karel Petrzik, Sára Brázdová, Krzysztof Krawczyk

**Affiliations:** 1 Biology Centre, Department of Plant Virology, Institute of Plant Molecular Biology, Czech Academy of Sciences, Branišovská 31, 370 05 České Budějovice, Czech Republic; sara.brazdova@umbr.cas.cz; 2 Department of Molecular Biology and Biotechnology, Institute of Plant Protection-National Research Institute, Władislawa Węgorka 20, 60-318 Poznań, Poland; k.krawczyk222@gmail.com

**Keywords:** complete genome, bonnellvirus, sortsnevirus, cronosvirus, kayfunavirus, winklervirus, myovirus

## Abstract

*Kosakonia cowanii* (syn. *Enterobacter cowanii*) is a highly competitive bacterium that lives with plant, insect, fish, bird, and human organisms. It is pathogenic on some plants and an opportunistic pathogen of human. Nine novel viruses that lyse plant pathogenic strains and/or human strains of *K. cowanii* were isolated, sequenced, and characterized. Kc166A is a novel kayfunavirus, Kc261 is a novel bonnellvirus, and Kc318 is a new cronosvirus (all *Autographiviridae*). Kc237 is a new sortsnevirus, but Kc166B and Kc283 are members of new genera within *Podoviridae*. Kc304 is a new winklervirus, and Kc263 and Kc305 are new myoviruses. The viruses differ in host specificity, plaque phenotype, and lysis kinetics. Some of them should be suitable also as pathogen control agents.

## 1. Introduction

*Kosakonia cowanii* is a recently reclassified bacterial species [1] within the Enterobacteriaceae family, previously known as *Enterobacter cowanii* [2]. It is a rod-shaped, motile, Gram-negative, facultative anaerobic bacterium, commonly present in soil and water [3] as well as in body environments of plants, animals, and human. Due to its ability to live in different environments and conditions, the bacterium is highly competitive environmentally and has huge metabolic potential. In water, wastewater, and sewage, *K. cowanii* has been identified as a component of biofilm-forming organisms [4]. In plants, *K. cowanii* has been found to be endophytic in alfalfa (*Medicago sativa*, Fabaceae) nodules [5] and in *Artemisia nilagirica* (Asteraceae), a traditional medicinal plant in Asia [6]. In animals, it has been found to inhabit the gut of the tropical *Anopheles gambiae* mosquito [7], as well as the gut of bees (*Apis mellifera mellifera*) hibernating under snow [8]. It was isolated from the intestine of Atlantic codfish (*Gadus morhua*) from the subpolar White Sea [9] and from nasal aperture of kea parrot (*Nestor notabilis*). In human, several strains of *K. cowanii* and *K. radicincitans* species have been isolated from clinical samples, including of blood, urine, bile, and sputum [2,10,11].

The bacterium is thought to be pathogenic primarily on plants, although it has been recognized as a component of the phylloplane of healthy plants [12]. It has been reported as a real pathogen of eucalyptus [13], woody plants [14], onion [15], and soybean [16]. Rare cases of opportunistic human infection have been reported; *K. cowanii* was identified as causative agent of rhabdomyolysis and bacteremia related to a rose thorn prick [17] and recently as a cause of acute cholecystitis in human [10]. On the other hand, co-inoculation of alfalfa plant with nonrhizobial bacteria *Klebsiella* sp. and *K. cowanii* and rhizobial *Sinorhizobium meliloti* bacteria has been observed to have plant-growth-promoting effect under conditions of salinity stress [5]. 

*K. cowanii* could have adverse as well as beneficial effects in human beings. In the interest of health protection, it will be advantageous to specifically control its presence in some environments where adverse effects of *K. cowanii* prevail. To date, no lytic phages as natural antagonists have been described for this bacterium, although more than 767 phages have been detected in Enterobacteriaceae generally, and 12 phages in *Enterobacter* sp. (NCBI.nlm.nih.gov accessed on 19 March 2021). In this paper, we describe nine novel bacteriophages (viruses) that lyse *K. cowanii*, some very quickly and with large burst size.

## 2. Materials and Methods

*K. cowanii* strains were isolated from symptomatic soybean leaves collected in Poland having tiny brown spots surrounded by a yellow halo [16]. Isolate from human patient with cholecystitis (hereinafter denoted as Hamburg strain) was a gift of Dr. Benjamin Berinson, Institute for Medical Microbiology, Virology and Hygiene, University Medical Centre Hamburg-Eppendorf, Hamburg, Germany. Type strain DSM 18,146 of *K. cowanii* was obtained from the DSMZ collection, Braunschweig, Germany. Soil samples for phage screening were collected from various locations and environments in the Czech Republic.

The bacteria were cultivated in tryptic soya broth (Merck) at 27 °C (the human strains at 37 °C) and plated in 0.45% soft agar onto solid medium in Petri dishes. Environmental phage samples from our collection were applied as 1 μL drops onto the plated culture and cultivated for 16 h at 27 °C. Clear zone formation on the plate then indicated presence of a specific lytic phage in the sample. More than 300 soil samples were tested in this way, and phages were recognized in nine samples. Phages from clear zones were collected into phage TSM buffer (10 mM Tris-HCl, pH 7.4, 150 mM NaCl, and 10 mM MgSO_4_), diluted, plated in soft agar with exponentially growing *K. cowanii* strain 045 and/or Hamburg strain cells, then cultivated. The plaques passage was repeated five times for each phage. For phage purification purposes, 200 mL of *K. cowanii* 045 or Hamburg strain culture 1 day old was inoculated with distinct phage and lysed 2 days with slow rotation at 27 °C or 37 °C, respectively. The cells were treated with 1% of chloroform added to the lysis culture, after 15 min the cells were centrifuged, and the virus was precipitated from the supernatant in the presence of 4% NaCl and 10% polyethylene glycol 6000 for 16 h. Sediment obtained after centrifugation at 15,000× *g* and 4 °C for 20 min was dissolved in TSM buffer, and the virus was purified by 3 h centrifugation at 112,000× *g* in an SW 28 rotor through a 30% sucrose cushion. 

Purified viruses were negatively stained with 2% (*w*/*v*) uranyl acetate and examined with a JEOL 1010 transmission electron microscope operating at 80 kV. 

Host specificity of the novel viruses was determined by spotting 1 µL of purified phage on soft agar layer containing putative host, cultivating for 16 h at 27 °C (at 37 °C with the human strains), then evaluating. 

For one-step growth curves, bacterial cultures were grown until OD_600_ = 0.3 (about 10^8^ CFU/mL) and inoculated with viruses to give multiplicity of infection > 1. Adsorption was for 10 min at 27 °C, followed by incubation on a shaker at the same temperature. Samples were taken every 20 min, diluted, then plated onto lawns of sensitive *K. cowanii* strain using the double agar overlay assay.

Kinetics of host cell lysis in liquid environment was evaluated in 100 µL volume of exponentially grown host cells and 10^6^ PFU of distinct phage and then evaluated in intervals at A_405_ nm. The experiment was performed independently three times.

DNA was extracted from the purified viruses after RNase, DNase, and proteinase K treatment as described previously for Arthronema phage [18] and then sequenced on the Illumina platform by Neogen (Cambridge, UK). Distinct reads were demultiplexed, adapter-trimmed, then assembled de novo in CLC Genomics Workbench 8.5.1. software (Qiagen, Hilden, Germany). The contigs were manually inspected and ambiguities were corrected after Sanger sequencing with specific primers. Corrected sequences were annotated in RAST [19]. HHpred was used for identification of distantly related proteins [20]. Sequence comparisons and phylogenetic analyses were performed in MEGA X [21]. Dot plot analysis was performed on concatenated complete genomes of selected bacterial viruses in the Gepard DotPlot program [22] with default parameter settings (word length = 10 and window size = 0).

The sequences were aligned by ClustalW with gap opening penalty = 10 and gap extension penalty = 0.10. Phylogenetic analysis was performed using MEGA X with the JTT model and confirmed by 1000 bootstrap replicates.

## 3. Results

### 3.1. Virus Identification

**Figure 1:**
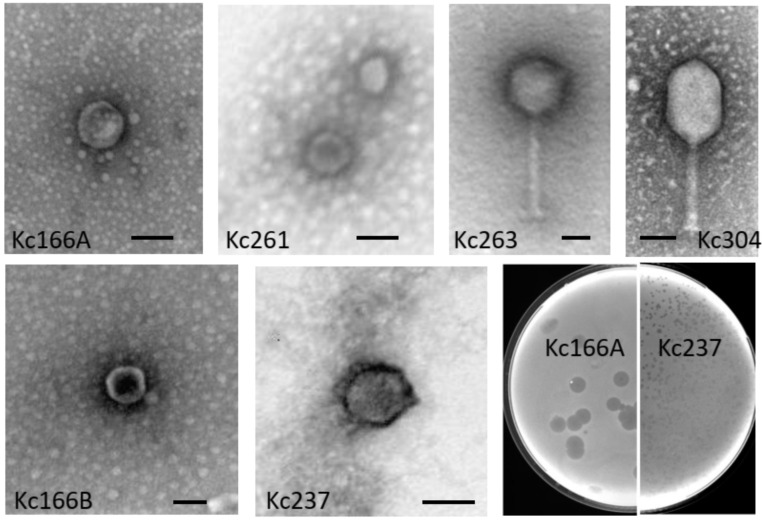
Morphology of the viruses. Purified viruses were stained with uranyl acetate and observed by JEOL 1010 electron microscope. Bars represent 50 nm. Plaque size of Kc166A and Kc237 viruses on 0.45% agar after 16 h incubation.

Six different viruses were identified in our soil samples using *K. cowanii* 042 and 045 as selection hosts. They were designated as Kc166A, Kc166B, Kc237, Kc261, Kc283, and Kc318. An additional three viruses (Kc263, Kc304, and Kc305) were identified using *K. cowanii* Hamburg strain. By electron microscopy after negative staining with uranyl acetate, isometric particles about 50 nm in diameter without apparent tail were observed for Kc166A, Kc261, and Kc318 viruses. Isometric particles about 60 nm in diameter with collar and short tail resembling viruses of the Podoviridae family were observed for Kc166B and Kc237 viruses. The Kc263, Kc304, and Kc305 viruses have particles with tails resembling those of myoviruses, the Kc263 has an isometric head 96 nm in diameter and tail of 175 nm with visible fibers, while Kc304 has an elongated head 70 × 107 nm and tail of size about 115 nm (Figure 1). Kc166A was the only virus producing large plaques (4 mm in 0.45% soft agar after cultivation overnight) with opaque halos. Such halo indicates that bacteriophage particles could contain enzymes degrading the lipopolysaccharides of bacterial cell wall [23]. All other viruses formed small plaques up to 1 mm in diameter under the same conditions.

### 3.2. Host Specificity

Host specificity was evaluated by spot test on soft agar with each given bacterial strain. Kc263 is the only virus that formed plaques on all nine plant strains as well as on both human strains of *K. cowanii*. On the other hand, Kc261 lysed only *K. cowanii* strain 042 from soybean leaves, and Kc305 was specific only for the Hamburg strain of *K. cowanii* from human bile. Kc166A, Kc166B, Kc237, and Kc261 lysed the plant strains, but not the human strains, while Kc263, Kc283, Kc304, and Kc318 lysed different strains from both sources (Table 1).

### 3.3. Growth Characteristics

The viruses completely lysed the host cells within 1–2 h. The latent period of the viral infection was longest (ca 60 min) for putative bonnellvirus Kc261 and shortest (30 min) for putative kayfunavirus Kc166A. The maximal phage progeny formation occurred 120 min after infection for Kc261 and after 90 min for Kc166A. Calculated burst size was about 80 particles per infected cell for Kc261 and about 180 particles/cell for Kc166A. By comparison, latent period and burst size of kayfunavirus Escherichia virus ZG49 are 9 min and 150 PFU/cell [24]. The latent period of the putative cronosvirus Kc318 (40 min) was much longer than that for Cronobacter Dev-CD-23823 and Cronobacter Dev-CT57 cronosviruses, which are 15 and 20 min, respectively, and also the burst size of Kc318 (90 particles) was smaller than those for the Cronobacter viruses (111 and 180 particles, respectively) [25]. The latent period of putative sortsneviruses Kc166B and Kc237 was 40 min, and burst size 100 PFU/cell.

**Figure 2:**
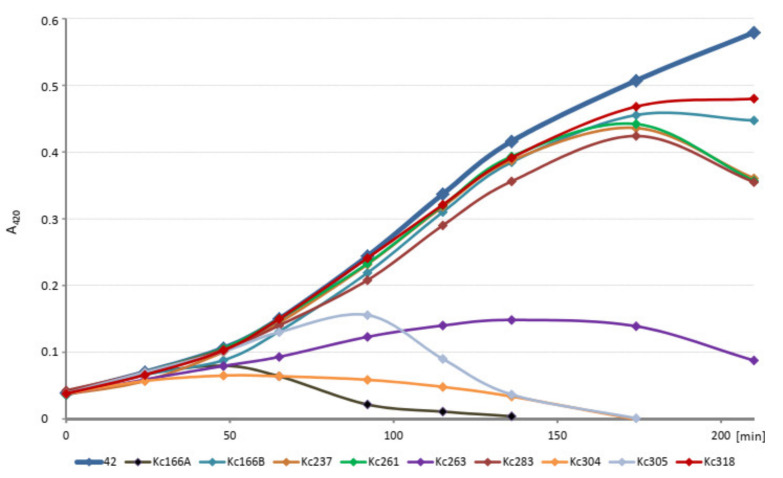
Kinetics of host cell lysis in liquid environment tested in 100 µL of host culture inoculated with 10^6^ each of phages and evaluated at A_420_ nm. Absorbance of the uninfected host is in bold.

The kinetics of host cell lysis in liquid environment was tested in 100 µL volume of exponentially growing host cells in ELISA plate and 10^6^ each of phages and evaluated in intervals for 4 h at A_420_ nm. The initial amounts of the host cells (strains 042, 045, and Hamburg, respectively) were diluted to identical values. All viruses inhibited growth of the host *K. cowanii* strain, but Kc166A virus did so most effectively and Kc318 virus did so least effectively (Figure 2).

### 3.4. Genome Characteristics of Kc261, Kc318 and Kc166A (Autographiviridae)

The genome of Kosakonia Kc261 virus is 42,203 bp long and with G+C content of 55.4%. All 54 predicted genes are present on a single strand (Figure 2). The genome arrangement and 87.2% nt identity with Escherichia phage usur (accession number MN850624) support classification of the virus in the genus *Bonnellvirus*, *Autographiviridae*. Furthermore, the structural major capsid protein (mCP) of Kosakonia virus Kc261 is almost 98% identical to the mCP of bonnellviruses (Table 2). Most probably, it is a novel virus within this genus. Kc261 has very narrow host range, as nine *K. cowanii* strains isolated from symptomatic soybean plants and two human strains were tested for host range, but Kc261 lysed only *K. cowanii* strain 042. Phage endolysin motifs were recognized on protein encoded by the ORF53 and should be the key enzyme of the Kc261´s lysis system. Unfortunately, to date, there are no data regarding host range of the other bonnellviruses that can be used for comparison purposes. The genome sequence of Kc261 was deposited in GenBank under AC: MW250275.

**Figure 3:**
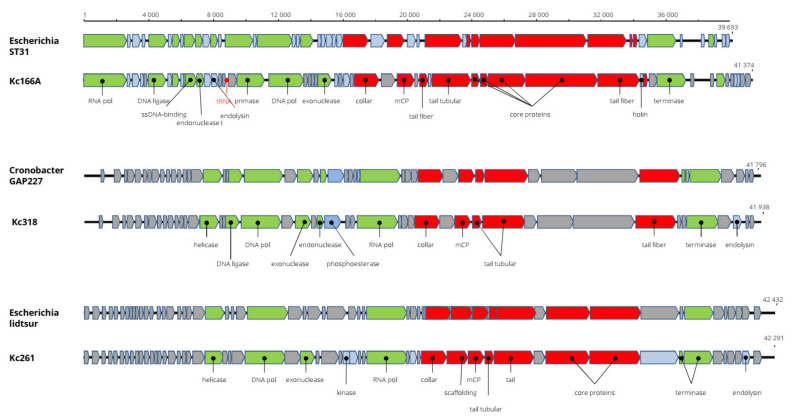
Genome organization of Kc166A, Kc318, and Kc261 in comparison with the closely related viruses. Here and in the next figures, the structural proteins are marked red, replication-associated proteins are light green, phage metabolism proteins are blue, proteins with regulatory functions are marked violet, hypothetical proteins with unknown functions are gray, and positions of tRNAs are marked red. All genes are drawn to scale.

The complete genome of Kosakonia Ks318 virus is 41,938 bp long and with G+C content of 55.5%. All the predicted 47 putative genes are arranged on a single strand. The total genome nt sequence reveals 91.3% identity with Cronobacter sakazakii phage GAP227 (accession number NC_020078, genus *Cronosvirus*, *Autographiviridae*) and more than 95% identity in aa sequence of most encoded proteins. The sequence differences are located in small hypothetical proteins before a putative DNA helicase gene and after locus 22, where a putative homing endonuclease is predicted in the Kc318 genome (Figure 3). We propose the name Kosakonia virus K318 (Kc318) for this virus and that Kc318 should be a novel virus in the genus *Cronosvirus*. The Kc318 lysed eight of nine *K. cowanii* plant isolates and also one human isolate. ORF45 encodes phage endolysin, which represents the phage lysis system. The complete genome sequence is in GenBank under accession number MW250276. The genome of Kosakonia virus Kc166A is 41,374 bp long and has G+C content of 52.7%. The 55 predicted ORFs are present on a single strand with a cluster of replication-associated proteins and a cluster of structural proteins. The genome arrangement is highly similar to that in viruses of the genus *Kayfunavirus* (*Studiervirinae*, *Autographiviridae*), and running a BLASTN search with whole genomes revealed that Escherichia phage ST31 is the most similar virus, with 68.8% nt identity. The Kc166A virus genome is the largest of all kayfunaviruses, which heretofore have reported genomes 39,252–40,792 nt long and with G+C content 49.7%–53.2% [26]. A pseudo-tRNA gene was predicted in a position similar to where such motif was predicted in some kayfunaviruses (Escherichia phage DY1, Escherichia phage ST31, and Escherichia phage LM33_P1). At present, there are about 30 viruses classified as members of this genus. In an mCP comparison, there is 96% aa identity with the corresponding gene of Cronobacter phage GW1. The Kc166A virus produced large plaques on test host strains, which implies a presence of enzymes degrading the lipopolysaccharides of the bacterial cell wall. Nevertheless, no such enzymes were found on the genome, and explanation for such feature must be sought elsewhere. Unlike previous viruses, in addition to phage endolysin, the holin gene, which can improve lytic abilities of the phage, was identified in the Ks166A genome. The genome sequence of Kc166A was deposited in GenBank under AC:MW258709. 

**Figure 4:**
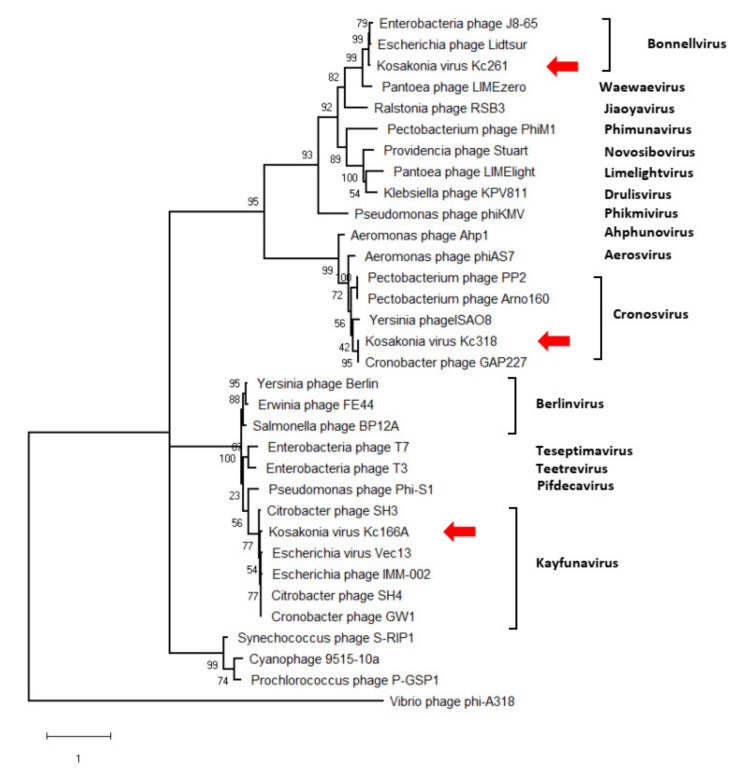
Maximum-likelihood phylogenetic tree computed on mCP amino acid sequences. Bootstrap values after 1000 repetitions are shown on the branches.

The genomes’ arrangement similarity and their sequence identity unequivocally classify the three viruses as distinct genera in *Autographiviridae*. Major capsid protein gene was identified in all three viruses, and a maximum-likelihood phylogenetic tree was computed with mCP amino acid sequences of selected autographiviruses. In this tree, Kc166A virus was classified within kayfunaviruses, Kc261 virus in genus *Bonnellvirus*, and Kc318 virus within genus *Cronosvirus* (Figure 4).

### 3.5. Genome Characteristics of Kc166B, Kc237 and Kc283 (Podoviridae)

**Figure 5:**
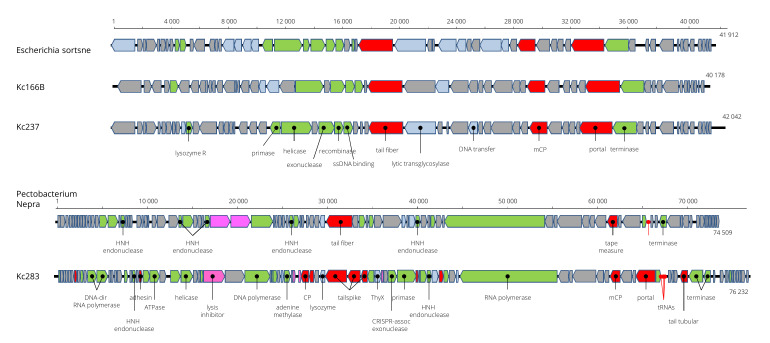
Genome arrangement of Kosakonia viruses Kc166B and Kc237 in comparison to Escherichia phage Sortsne and Pectobacterium phage Nepra.

Viruses designated as Kc166B and Kc237 have isometric particles with short tail similar to that of podoviruses (Figure 1). Also, the genome arrangement of 54 and 57 predicted genes, respectively, coded on both strands resembles the genome arrangement of podoviruses from genera *Sortsnevirus* and *Giessenvirus* (Figure 5). The complete genome of Kc166B is 40,785 bp long and has G+C content of 57.4%. Escherichia phage C130_2 (AC No. NC_048067) and Escherichia phage Sortsne (AC No. NC_048178) were 53.5% and 48% identical, respectively, when complete nt sequences were aligned in MUSCLE. The complete genome of Kc237 virus is 40,897 bp and has G+C content of 59.4%. The genome of Klebsiella phage IME279 was recognized as the most similar genome, with 72.1% nt identity. The host specificity of the viruses seems to be very narrow, as Kc166B and Kc237 lysed *K. cowanii* strains 042 and 045 only. A phage lysozyme R gene more probably represents the lysis system in both viruses. The genomes are deposited in GenBank under AC MW258711 and MW258710, respectively. Kosakonia virus Kc283 has also podoviral-like particles, but a much larger genome of 76,232 bp than the previous two viruses. Its G+C molar content is 46.9%. It encodes for 91 ORFs on both strands, and three tRNAs for Asn, Tyr, and Gln. The genome has a close relationship to unclassified podoviruses, with Pectobacterium phage Nepra, Pectobacterium phage CB1, and Pectobacterium phage Possum as the most similar, with 74.6%, 74.4%, and 69.0% nt sequence identity, respectively. As in the related viruses, homing endonucleases were identified on the genomes (products of ORF19 and ORF58) and putative recombinase A gene adjacent at ORF57. Furthermore, a crossover junction endodeoxyribonuclease RuvC gene that resolves Holliday junction intermediates in genetic recombination was predicted on the viral genome as a product of ORF61. Phage lysozyme R represents the lysis system of this virus. The sequence was deposited in GenBank under AC No: MZ348421.

**Figure 6:**
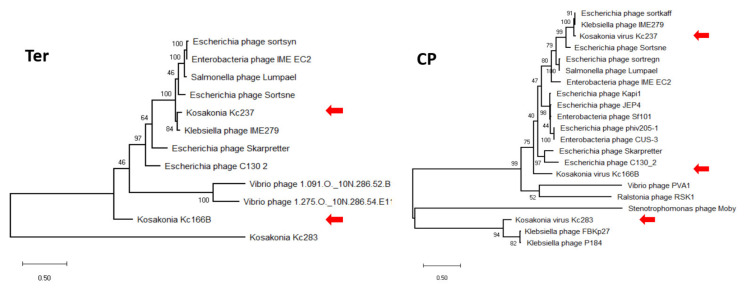
Maximum-likelihood phylogenetic tree computed on mCP (**a**) and terminase (**b**) aa sequences.

**Figure 7:**
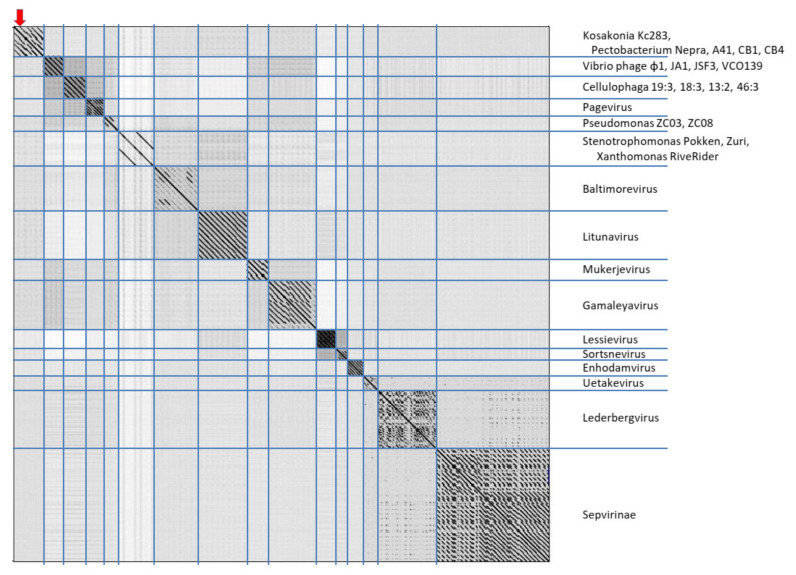
Dot plot of concatenated podovirus complete sequences generated in Gepard [22] with default parameters setting (word length = 10 and window size = 0). Position of Kc283 is marked.

In maximum-likelihood trees computed according to mCP and terminase aa sequences (Figure 6), Kc237 virus was classified together with Escherichia phage Sortsne and Klebsiella phage IME279 (both sortsneviruses). Kc166B is in both analyses distantly related to sortsneviruses, giessenviruses, and skarpretterviruses, and more probably it is a member of a novel genus. To clarify the taxonomic position of Kc283, a dot plot of concatenated complete genomes of podoviruses was created in Gepard [22]. In this plot, Kc283 revealed sequence homology with unclassified podoviruses Pectobacterium phage Nepra, A41, CB1, and CB4—which are all viruses with genomes of about 75 kbp and G+C content about 48%—but not with other podoviruses (Figure 7). More probably, this virus will be a member of a novel genus not yet established.

### 3.6. Genome Characteristics of Kc304, Kc305 and Kc263 (Myoviridae)

**Figure 8:**
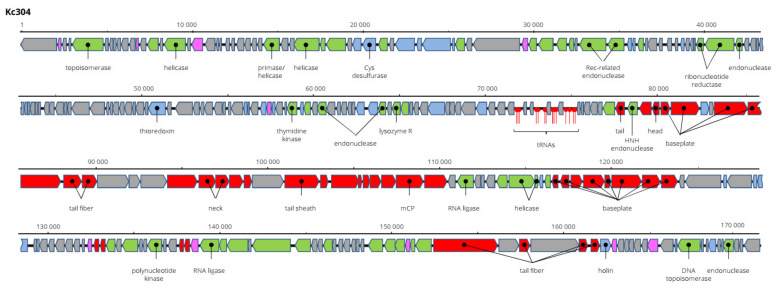
Genome structure and annotation of the Kc304 virus.

Kosakonia virus Kc304 has a genome 171,207 bp long and 94.2% nt identical to Serratia phage CH14 (*Myoviridae*, *Winklervirus*, NC_041996). Differences in the genomes are mainly due to the presence or absence of homing endonucleases on the genomes (Figure 8). If ignoring these elements, Kc304 could be considered an isolate of Serratia phage CH14. Fifteen tRNA genes were predicted on the Kc304 genome, while there are 16 tRNA genes on the Serratia phage CH14 genome. Two phage recombination-related endonucleases—uvsY.2 recombination protein and RuvA helicase—could be components of the putative recombination system of Kc304. Phage lysozyme R represents the lysis system of this virus. Kc304 lysed three out of nine strains of *K. cowanii* from plants and both isolates from human. The sequence was deposited in GenBank under AC No: MZ348424.

**Figure 9:**
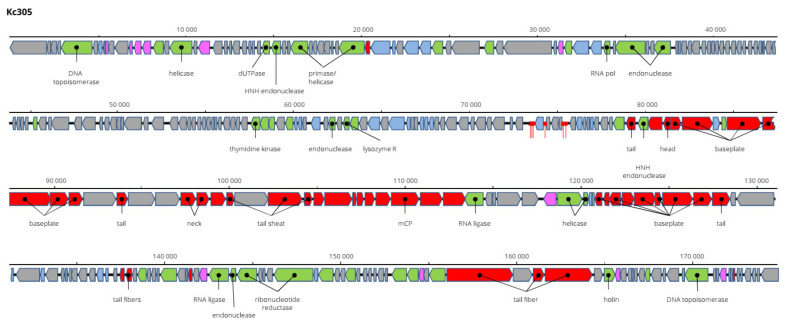
Genome structure and annotation of the Kc305 virus.

**Figure 10:**
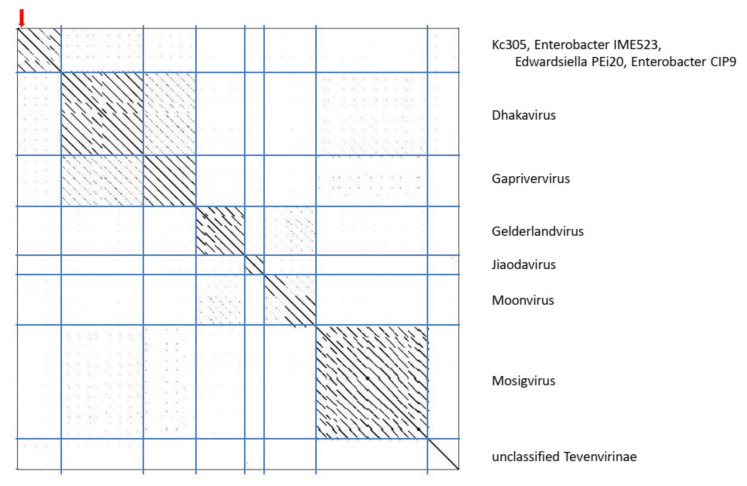
Dot plot of concatenated Kc305 and related myoviruses.

The genome of Kc305 is 174,783 bp long and with G+C molar content of 40%. Annotated on the genome in both orientations were 295 predicted ORFs and six tRNA genes (Figure 9). No integrase was identified on the Kc304 and Kc305 genomes, and these viruses more probably did not exist as integrated into the host genome, but several proteins involved in recombination/repair function, such as recombination-related endonucleases, uvsY.2 protein, and ssDNA-binding proteins, were identified there. In dot plot analysis of the complete genome (Figure 10), Kc305 is in a group with Enterobacter phage vB EhoM-IME523 (86.6% nt sequence identity), Edwardsiella phage Pei20 (69.3% nt identity), and Enterobacter phage vB_EclM_CIP9 (84.4% nt identity) [27], all of which are unclassified Tevenvirinae (*Myoviridae*), close to dhakaviruses and gapriverviruses. *K. cowanii* strain Hamburg is the only host of this virus. The sequence was deposited in GenBank under AC No: MZ348423.

**Figure 11:**
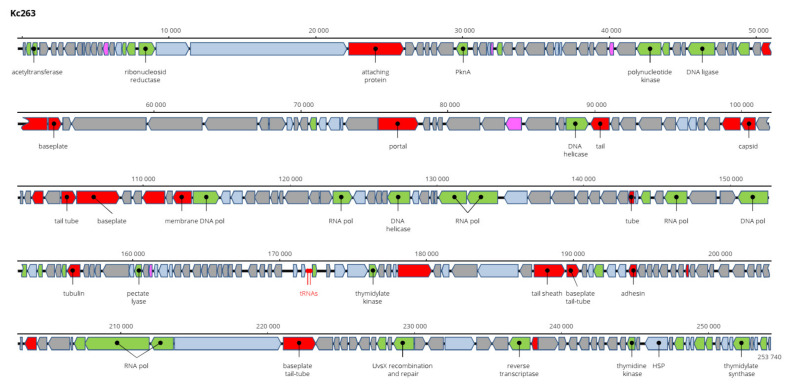
Genome structure and annotation of the Kc263 virus.

The genome of Kc263 is the largest of all viruses of *K. cowanii* found and sequenced. It is 253,740 bp long and encodes 259 ORFs on both strands and two tRNA genes (Figure 11). Aeromonas phage PS1, Klebsiella phage N1M1, and Pseudomonas phage OBP (all *Myoviridae* members) are related viruses, but with only about 50% nt sequence identity. Its taxonomic position is not clear, and more probably it will be a member of a novel genus. This virus has the widest range of hosts, as it lysed all 11 tested *K. cowanii* strains. The sequence was deposited in GenBank under AC No: MZ348422.

## 4. Discussion

The *K. cowanii* species was established as comb. nov. of *K. cowanii*, *E. radicincitans*, *E. oryzae*, and *E. arachidis* and can be variable in its biological features despite that the molecular classification based on concatenated gyrB, rpoB, infB, and atpD gene sequences bunched all the old species into one cluster [1]. Various *Kosakonia* spp. (*K. arachidis*, *K. oryzae*, K. *oryziphilus*, *K. sacchari*, *K. pseudocacchari*, and *K. oryzendophyticus*) are known as plant-promoting bacteria providing for better plant survival under salinity stress, cold stress [28,29], or stress caused by bioavailable metals [30], as well as for stimulating plant growth and biomass [31] or providing antagonistic activity toward pathogenic fungi [32]. At the same time, *K. cowanii* is recognized to be a real plant pathogen of soybeans and melon fruit [16,33]. The variety of hosts (insects, plants, fish, birds, and human) and environments indicates the high metabolic capability of *K. cowanii*. From this point of view, *K. cowanii* seems to be a very promising bacteria for biotechnology purposes [34]. On the other hand, when the genome of *K. cowanii* type strain 888-76^T^ was sequenced, 23 genes responsible for antibiotic resistance as well as 262 genes predicted to be virulence genes were identified [3]. No natural antagonist of this bacterium has been known until now. We have described here a significant number of nine novel viruses that infect different plant and human strains of *K. cowanii*. All the viruses differ from known viruses and thus constitute novel species or even novel genera. The novel bonnellvirus and novel sortsnevirus have narrow host ranges, but the novel kayfunavirus and novel cronosvirus lyse plant as well as human *K. cowanii* strains and could be promising for use as a bio-curative tool in either plant protection or in medicine.

Bacteriophages have been found to be effective for control of several phytopathogenic bacteria such as *Erwinia* spp., *Xanthomonas* spp., *Pseudomonas* spp., *Ralstonia solanacearum*, and *Streptomyces scabies* [35]. They are usually used as a mixture of phages to overcome the risk of reduction in efficacy due to spontaneous surface receptor mutations that prevent the adsorption of phages on their bacterial hosts and lead to bacterial resistance [36]. The control efficacy of phages in the fields and orchards is also limited by environmental and weather conditions, as phages are inactivated by heat, UV irradiation, desiccation, and osmotic stress, and could be washed off by rain [37]. We therefore believe that the best place for application of bacteriophages in agronomy will be seed pretreatment before sowing or planting, where the conditions could be better controlled [38], or in product treatment before distribution to the retail chain, where phages can suppress the development of bacteriosis and extend the shelf life of products. There are also data describing the penetration of phages from the soil and their translocation ability in plant tissue [39,40], which open the door to the administration of curative phages by controlled irrigation or via hydroponic growth medium. Recently, *K. cowanii* and *K. radicincitans* species were documented to be facultative human pathogens causing bacteremia or cholecystitis [1,10,11,41]. Although such human infections seem to be rare, it is also probable they have been underestimated inasmuch as the pathogen could be misidentified as *Enterobacter* sp. [10,11]. No preclinical trials have been performed for *Kosakonia* sp. or *Enterobacter* sp., but such studies about the in vivo application of phages have been published for related *Klebsiella pneumoniae*, *Salmonella enteritidis*, or *Escherichia coli* [42]. In many cases, the administration of a cocktail of phages has usually been shown to be better than a monophage therapy [42]. Five viruses that lyse one or the other or both strains of *K. cowanii* from a human and described in this paper are the first tools available for such tests. Although the Kc263 novel myovirus has the widest host range, as it lysed all tested *K. cowanii* strains, a protein highly homologous to UvsX, which is involved in DNA recombination and repair [43], most probably disqualifies it for bio-curative purposes. In addition, there are a number of genes of unclear origin and function in all viruses found that still need to be clarified to use the viruses safely.

## 5. Conclusions

Nine viruses have been found to lyse plant and/or human strains of *Kosakonia cowanii*. The new viruses were completely sequenced and analyzed. Three were classified in Podoviridae, three in Autographiviridae, and three in Myoviridae. Kc166B and Kc283 viruses more probably represent species within genera among Podoviridae that are not yet established.

## Figures and Tables

**Table 1:** Host specificity of Kosakonia viruses was tested on nine plant isolates and two human isolates of *K. cowanii*. Plaque formation is marked “+”, absence of plaque is marked “-“.

Virus	*K. cowanii* Strains											Classification	
	039	042	045	048	051	056	059	062	063	18146	Hamburg		
	plant strains									human strains			
**Kc166A**	-	+	+	-	+	+	+	+	+	-	-	Kayfunavirus	*Autographiviridae*
**Kc261**	-	+	-	-	-	-	-	-	-	-	-	Bonnellvirus	*Autographiviridae*
**Kc318**	+	-	+	-	+	+	+	+	+	+	-	Cronosvirus	*Autographiviridae*
**Kc166B**	-	+	+	-	-	-	-	-	-	-	-	novel genus	*Podoviridae*
**Kc237**	-	+	+	-	-	-	-	-	-	-	-	Sortsnevirus	*Podoviridae*
**Kc283**	-	+	-	+	-	-	+	-	-	-	+	novel genus	*Podoviridae*
**Kc263**	+	+	+	+	+	+	+	+	+	+	+	novel genus	*Myoviridae*
**Kc304**	+	-	+	-	-	-	-	-	+	+	+	Winklervirus	*Myoviridae*
**Kc305**	-	-	-	-	-	-	-	-	-	-	+	Myovirus	*Myoviridae*

**Table 2:** Genome nucleotide sequence percentage identities of bonnellviruses (above diagonal), and major capsid protein (mCP) aa sequence identities (below diagonal).

	Smaasur MN850625	Ent.J8-65 NC_025445	Altidsur MN850568	Aldrigsur MN850592	Mellemsur MN850570	Megetsur MN850608	Glasur MN850583	Forsur MN850617	Usur MN850624	Kc261 MW250275	Lidtsur NC_048177
**smaasur**		93.8	74.2	73.7	75.2	75.2	73.0	73.7	74.4	73.6	72.7
**Ent. J8-65**	99.4		74.2	73.9	75.1	75.1	72.9	73.5	74.2	73.4	71.9
**altidsur**	93.3	93.3		97.1	88.2	88.8	80.4	80.3	80.3	82.5	70.5
**aldrigsur**	93.3	93.3	99.7		87.9	88.6	80.6	80.4	80.5	82.6	70.1
**mellemsur**	92.4	92.4	99.1	98.8		95.9	79.9	81.3	81.0	82.2	70.4
**megetsur**	93.3	93.3	100.0	99.7	99.1		80.5	80.9	81.0	82.7	70.8
**glasur**	94.2	94.2	95.2	95.2	94.2	95.2		94.6	90.8	86.3	70.3
**forsur**	94.2	94.2	95.2	95.2	94.2	95.2	100.0		92.7	86.6	70.1
**usur**	93.9	93.9	94.8	94.8	93.9	94.8	99.4	99.4		87.2	69.6
**Kc261**	93.6	93.6	97.6	97.6	96.7	97.6	97.0	97.0	97.3		71.0
**lidtsur**	94.5	95.2	94.5	94.5	93.6	94.5	96.4	96.4	96.1	95.8	

## Data Availability

All sequence data is deposited in GenBank.
